# Effects of whole-grain cereals on fecal microbiota and short-chain fatty acids in dogs: a comparison of rye, oats and wheat

**DOI:** 10.1038/s41598-023-37975-4

**Published:** 2023-07-05

**Authors:** Hanna Palmqvist, Katja Höglund, Sara Ringmark, Torbjörn Lundh, Johan Dicksved

**Affiliations:** 1 Department of Animal Nutrition and Management, Faculty of Veterinary Medicine and Animal Science, Swedish University of Agricultural Sciences, Uppsala, Sweden; 2 Department of Anatomy, Physiology and Biochemistry, Faculty of Veterinary Medicine and Animal Science, Swedish University of Agricultural Sciences, Uppsala, Sweden

**Keywords:** Animal physiology, Microbiota

## Abstract

Dietary fiber in dog food is reported to promote healthy gut microbiota, but few studies have investigated the effects of whole-grain cereals, which contain a variety of fiber types and other bioactive compounds. The aim of the present study was to compare the effects of diets containing whole-grain rye (RYE), oats (OAT) and wheat (WHE) on fecal microbiota and short-chain fatty acid production. Eighteen dogs were fed three experimental diets, each for four weeks, in a cross-over design. Fecal samples were collected at the end of each diet period. Analysis of 16S rRNA gene amplicons showed that family *Lachnospiraceae* and genus *Bacteroides* were the gut microbial groups most affected by diet, with lowest relative abundance following consumption of RYE and a trend for a corresponding increase in genus *Prevotella*_9. Fecal acetate and propionate concentrations were higher after consumption of RYE compared with OAT. In conclusion, rye had the strongest effect on gut microbiota and short-chain fatty acids, although the implications for dog gut health are not yet elucidated.

## Introduction

Several studies in dogs have investigated potential promotion of a healthy gut microbiota by specific dietary fiber types^1–3^, fiber-rich food by-products, or fiber blends added to the food^4–6^. However, few studies have investigated how gut microbiota composition and function are affected by addition of fiber as part of the whole-grain component in dog food^7,8^. Whole grains contain several bioactive compounds, such as tocols, phytosterols, alkylresorcinols and other phenolic compounds, which can have additive or even synergistic effects with the gut microbiota on host health according to studies in humans and pigs^9,10^. Rye, wheat, oats and barley are the most commonly grown grain crops in the Nordic countries and all contain different combinations of dietary fiber and bioactive compounds. Rye and wheat grains have a high content of arabinoxylan, while oat and barley grains are high in β-glucans^11^. Rye has the highest content of fructan, phytosterols and alkylresorcinols and, together with wheat, the highest content of tocols^10^. Hence, it is likely that these grain types have different effects on the gut microbiota.

Arabinoxylan, β-glucan and fructans are soluble dietary fibers readily fermented by the gut microbiota, which in the process produce short-chain fatty acids (SCFA), primarily acetate, propionate and butyrate^12^. These SCFA serve as substrates in the metabolism and interact with receptors on the intestinal cells, which then release metabolic hormones^13–15^. Furthermore, SCFA are acidic, hence lowering the pH in colon. The low pH favors potentially beneficial bacteria, such as *Bifidobacterium* and *Lactobacillus*^16,17^, and restricts proliferation of bacteria associated with gastrointestinal disease in dogs, such as *Clostridium perfringens* and *Escherichia coli*^18,19^. The absolute and relative amount of the different SCFA produced during fermentation of dietary fiber depends on the species composition of microbiota as well as the type and solubility of the dietary fiber^20^.

We have previously compared the effects of whole-grain rye and refined wheat on the fecal microbiota in dogs^21^. In that study, we observed an increase in genus *Prevotella* relative to baseline values when rye was included as 50% of dry matter in the diet. No such effect was observed when the dogs were fed a refined wheat diet. Some studies in humans have reported beneficial effects of whole grain in the diet on host metabolism, with or without major changes in gut microbiota^22–24^. However, as those studies compared whole-grain diets with refined cereal products, the results could not clarify whether differences observed were due to effects of the grain-specific fibers and bioactive compounds, or effects of a high-fiber versus a low-fiber diet. A few studies on humans have compared the effects of whole grains from different sources (mostly rye and wheat) on gut health-related parameters such as SCFA concentration and microbial community composition^25,26^. In dogs, such effects have been studied when feeding dietary oat groats or a number of other ancient grains^8^, but to our knowledge there is no previous comparative study on dogs fed diets including whole-grain wheat or rye. Therefore, the aim of this study was, to explore the effects of dietary inclusion of three different whole grain types (rye, oats and wheat) on fecal microbiota composition and SCFA production in dogs. Our hypothesis was that given the differences in fiber and bioactive component composition of the whole grains, they would promote different bacterial taxa and thus the microbial composition and their fermentative products would differ.

## Material and methods

This explorative, experimental, cross-over diet study was performed in August-December 2019. The dogs were privately owned and lived in their home environment throughout the study period. Examinations of the dogs and analyses were performed at the Swedish University of Agricultural Sciences in Uppsala, Sweden. The study was approved by the Ethics Committee for Animal Experiments, Uppsala, Sweden (Approval no. 5.8.18-18808/2017-7) and complied with ARRIVE guidelines^27^. Written informed consent was obtained from all dog owners before the start of the study. Information on the owners was handled in accordance with general European Union data protection regulations (Regulation (EU) 2016/679).

### Diets

Three extruded experimental diets, containing whole-grain flour of rye (RYE), wheat (WHE) or ground rolled oats (OAT), were produced by the commercial dog food producer Doggy AB, Vårgårda, Sweden (Table 1).

**Table 1:** Ingredients in the three experimental diets, expressed as % included.

Ingredient	Wheat diet	Oat diet	Rye diet
Wheat flour	25	–	–
Oat flour	–	25	–
Rye flour	–	–	25
Maize	15	15	15
Rice	11.7	12.5	10.8
Lignocellulose^a^	1.5	1.5	1.5
Dried chicken meal	29.7	29.9	30.4
Fresh chicken meat	5	5	5
Chicken stock^b^	3	3	3
Pork fat	7.5	6.4	7.6
Premix of minerals and vitamins^c^	1.7	1.7	1.7

^a^Source of insoluble fiber consisting of lignin, cellulose and hemicellulose.

^b^Source of flavor.

^c^Nutrients added per kg: Vitamin A (IE) 11,100, Vitamin D3 (IE) 1160, Vitamin E (mg) 299, Vitamin C (mg) 403, Thiamine B1 (mg) 2.9, Riboflavin B2 (mg) 4, Pyridoxine B6 (mg) 2, Niacin B3 (mg) 19.9, Pantothenic acid B5 (mg) 17.6, Biotin (mg) 0.2, Vitamin B12 (mg) 0.06, Folic acid (mg) 0.4, Copper(II) sulphate pentahydrate (mg) 23, Copper (mg) 6, Manganese(II) oxide/ manganese(III) oxide (mg) 9.6, Manganese (mg) 5.8, Zinc sulphate monohydrate (mg) 101, Zinc (mg) 36, Calcium iodate anhydrate (mg) 17.8, Iodine (mg) 1.8.

The diets were nutritionally complete and balanced according to FEDIAF guidelines^28^. The level of grain inclusion was set at 25%, as fed, based on previous results^21^. The diets were also composed to be similar in terms of protein and metabolizable energy content (Table 2).

**Table 2:** Chemical composition of the experimental diets, expressed as % of dry matter (DM) unless otherwise stated.

Item	Wheat diet	Oat diet	Rye diet
Dry matter	93.9	93.5	93.9
Gross energy (MJ/kg DM)	20.9	21.7	21.5
Metabolizable energy (MJ/kg DM)^a^	17.2	18.0	17.8
Organic matter	92.9	93.1	92.7
Crude protein	30.5	30.4	29.5
Crude fat	13.7	17.6	16.7
Crude fiber	1.9	1.5	1.8
Total dietary fiber	8.6	7.6	9.1
Soluble dietary fiber	1.4	2.0	1.7
Insoluble dietary fiber	7.2	5.6	7.4
Total starch	38.3	34.2	34.8
Resistant starch	0.2	0.2	0.3
Non-resistant starch	38.1	34.0	34.5

^a^Calculated according to NRC 2006^61^.

### Animals and study design

Healthy dogs were recruited among staff and students at the Swedish University of Agricultural Sciences and the University Animal Hospital in Uppsala, Sweden. This population was chosen as these dog owners could be expected to have high treatment compliance and competence to follow study instructions due to their competence within veterinary medicine and animal science. The criteria for inclusion were a minimum age of 12 months and a minimum body weight (BW) of 7 kg. Exclusion criteria were: antibiotic treatment within three months prior to the study, known intolerance or allergy to any of the experimental food ingredients, or history of sensitivity to diet change. Before the first experimental diet period, all dogs underwent a health assessment, which included a physical examination (all performed by the same veterinarian), routine hematology, serum biochemical analysis and urine analysis (standard dipstick chemistry test, urine specific gravity and protein/creatinine ratio). Dogs were excluded if there were any clear findings indicating systemic or organ-related disease or if they had a gastrointestinal reaction to the diets that affected their general condition (mild, transient alterations of fecal consistency and frequency were allowed).

The study was performed as a reduced 3 × 3 Latin square, in which each dog acted as their own control and with the diet orders WHE-OAT-RYE, OAT-RYE-WHE and RYE-WHE-OAT. The dogs were categorized by gender and size, anonymized and randomly divided into three groups. The groups were then randomly assigned a diet order. Pairs of dogs living in the same household were kept together and followed the same diet order. Each experimental diet was fed for a minimum of four weeks before sampling. A transition period of 4–7 days preceded each diet period. During the transition periods, the owners were instructed to mix the dog’s present food with the new diet in increasing amounts to allow the dog to adapt to the new diet. In order to make the starting conditions as similar as possible for all dogs they were all first fed WHE for three weeks, including a 4–7 day transition period, before starting the first experimental diet period.

All owners were blinded to the content of the diets. The owners were instructed to keep to the dog’s normal feeding routines and to weigh their dog on the same scale once a week. The start daily feed allowance was based on the amount of calories in each individual’s normal feed intake, calculated before the study. Daily caloric intake was adjusted to maintain original BW by increasing or decreasing the allowance by 5–25%, based on the level of BW change and then evaluating BW the following week. The owners were instructed to feed the experimental diet as the main source of energy, but treats were allowed as long as they were given in approximately the same amount during each diet period. However, owners were instructed to give nothing but the experimental diet during the last three days before sampling in each diet period.

At the end of each diet period, owners were asked to fill in a form with both closed and open questions concerning compliance to the instructions, as well as the wellbeing of the dog, during the diet period.

### Fecal sampling and handling

Fecal samples for microbial and SCFA analyses were collected once during one of the two last days of each experimental period. All samples was collected immediately after voiding. Samples were either placed in − 20 °C within 2 h from sampling, or kept chilled and placed in − 20° within 4 h from sampling. Samples were then stored for a maximum of seven months before analysis.

Analyses of the samples were performed by laboratory staff who were blinded to the diets.

### Microbiota analysis

For DNA extraction, 180–220 g of fecal matter were transferred to a sterile tube containing 0.3 g sterilized 0.1 mm zirconia/silica beads (Biospec products, Bartlesville, Oklahoma, USA), followed by addition of 1 ml InhibitEX buffer (Qiagen Gmbh, Hilden, Germany). The samples were vortexed and homogenized in a Precellys24 sample homogenizer (Bertin Technologies, Montigny-le-Bretonneux, France) at 6500 rpm for 2 × 60 s, to disrupt bacterial cell walls. A QIAamp Fast DNA Stool Mini Kit (Qiagen Gmbh, Hilden, Germany) was used according to the manufacturer’s protocol to isolate DNA. For generation of 16S rRNA gene amplicon libraries and sequencing, extracted samples were sent to Novogene (Tianjin, China).

### Library preparation and sequencing

The V3-V4 region of the 16S rRNA gene was amplified using the primers 341F (5-GTGCCAGCMGCCGCGGTAA-3) and 805R (5-ACTACHVGGGTATCTAATCC-3). Polymerase chain reactions (PCRs) were performed using Phusion^®^ High-Fidelity PCR Master Mix (New England Biolabs) and the amplicons generated were confirmed by gel electrophoresis, purified with Qiagen Gel Extraction Kit (Qiagen, Germany) and quantified using a Qubit^®^3.0 Fluorometer (Invitrogen, Thermo Fisher Scientific). The final library including barcodes and adaptors was generated with the NEBNext^®^ UltraTM DNA Library Prep Kit. The amplicons were then sequenced using Illumina sequencing (NovaSeq 6000) at Novogene (Beijing, China). The sequence data obtained were deposited in the Sequence Read Archive (SRA), under accession number PRJNA933568.

### Bioinformatics analysis

Paired-end reads were assigned to each sample based on their unique barcode. After truncating the barcode and primer sequence using FLASH (v1.2.71) paired-end sequences were merged^29^ and the raw data sequences were quality-filtered using QIIME (v1.7.02)^30,31^. Sequences were clustered into operational taxonomic units (OTUs) using Uparse software (Uparse v7.0.10013)^32^, where sequences with ≥ 97% homology were assigned to the same OTU. Representative sequences for each OTU were screened for further annotation. For each representative sequence, the Mothur software was applied to the SSU rRNA data in the SILVA Database for species annotation at each taxonomic rank^33,34^.

### Short-chain fatty acid analysis

Acetate, propionate, and butyrate were analyzed in 0.5 g fecal matter dissolved in 1 mL 5 mM H_2_SO_4_, as previously described^35^. The high-performance liquid chromatography system consisted of an Alliance 2795 separation module and a 2414 RI Detector (Waters Corp. Milford, MA, USA). ReproGel H 9µ 300 × 8 mm (Dr. A. Maisch, Ammerbuch, Germany) functioned as the separation column and a ReproGel H, 9µ 30 × 8 mm, was used as the pre-column.

### Chemical analysis of food

The dog food samples were analyzed without pre-drying. To determine dry matter (DM) content, the samples were dried at 103 °C for 16 h, and then placed in a desiccator to cool before weighing^36^. For ash determination, samples were incinerated at 550 °C for three hours and then cooled in a desiccator before weighing. The Kjeldahl method^37^ was applied to determine nitrogen content, using a 2020 digester and a 2400 Kjeltec analyzer (FOSS Analytical A/S, Hilleröd, Denmark). Crude protein was then calculated as N × 6.25. Crude fat was analyzed in accordance with Commission Directive EC/152/2009^38^, on a Soxtec extraction unit (FOSS Analytical A/S, Hilleröd, Denmark). Crude fiber was analyzed as previously described^39^. Gross energy (GE) was measured on a Parr isoperobol Bomb Calorimeter 6300 (Parr Instrument Company, Moline, Illinois, USA). Total dietary fiber (TDF) as well as soluble and insoluble dietary fiber were analyzed in accordance with AOAC 991.43 using a Total Dietary Fiber Assay Kit (Megazyme, Bray, Ireland), while resistant and non-resistant starch were analyzed according to AOAC 2002.02 using a Resistant Starch Assay Kit (Megazyme, Bray, Ireland).

### Statistical analysis

Effects of diet on general microbial community composition were analyzed by principal coordinate analysis (PCoA), based on Bray Curtis distance and including relative abundance data from all OTUs, using the software PAST^40^. A linear mixed effects model with treatment and treatment order as fixed effects and dog as random effect, together with continuous autoregression correlation of time per dog, was set up using the *nlme* package v3.1.157^41^ in R version 4.2.1^42^. The model was used for univariate analyses of genera and OTUs with mean relative abundance > 0.1% and with maximum 25% zero counts, and for data on diversity and SCFA. Models were checked for normality and homoscedasticity with residual plotting. Diversity data from Chao1 index and PD whole tree, as well as relative abundance data, were transformed by the natural logarithm before statistical analysis. A constant of 1 × 10^–5^ was added to genera and OTUs with zero counts in order to do the transformation. The *p*-values obtained for relative abundance were corrected for multiple testing using false discovery rates (FDR) according to Benjamini-Hochberg^43^. Comparisons of estimated marginal means were corrected using Tukey’s adjustment. Differences were considered significant if *p* ≤ 0.05.

## Results

### Participating dogs

Initially, 22 dogs were recruited. However, four dogs were excluded in agreement with their owners during acclimatization to the diets or early in the first experimental diet period due to problems with loose stools, signs of possible cutaneous adverse food reaction or palatability issues. All remaining 18 dogs were assessed as healthy before the first experimental diet period. The dogs were of 12 different dog breeds (2 dogs were of the same breed) and 5 mixed dog breeds. The mean ± SD age was 5.7 ± 2.6 years, mean ± SD body condition score (BCS) on a 9-point scale^44^ was 5.2 ± 0.6 and mean ± SD body weight (BW) was 18.4 ± 9.5 kg. There were three pairs of dogs living in the same household. The pairs were randomized to different groups. A detailed demographic description of all included dogs can be found in Supplementary Table S1.

### Outcome of the intervention

All dogs remained on the experimental diets throughout the whole study period. Body weight change (mean ± SD) from the start of the experimental part to last sampling was − 0.03 ± 0.50 kg for all dogs, while for the different diets it was: WHE: − 0.1 ± 0.41, OAT: 0.2 ± 0.38, RYE: − 0.1 ± 0.33 kg. Intake of TDF (mean ± SD), in gram per kg BW and day, in dogs on the different diets was: WHE: 1.0 ± 0.3, OAT: 0.9 ± 0.2, and RYE: 1.1 ± 0.3 g. There were no indications in the owners’ reports of any significant deviations regarding compliance with the diet instructions. All dogs completed the whole study except one dog, which died during the last diet period. Necropsy showed that the dog died for reasons not related to the study. Five dogs were treated with non-steroidal anti-inflammatory medication for at least one short period during the study, for reasons not related to the study. One dog was treated with a combined betametason and fucidic acid ointment for a localized skin infection during the last diet period. The infection had unknown etiology, but healed and did not recur after completion of the medical treatment, which ended three weeks prior to the last fecal sampling. The results from the analysis of that dog’s final fecal sample revealed that it did not deviate from the samples collected previously from that dog, and hence it was included in the statistical analysis.

### Microbiota analyses

The sequence analysis generated on average 112,113 (range: 46,162–126,364) sequences per sample. The most abundant genera in the sample set were: *Fusobacterium* (Fusobacteria), *Prevotella*_9 (Bacteroidetes), *Bacteroides* (Bacteroidetes), *Catenibacterium* (Firmicutes) and *Peptoclostridium* (Firmicutes).

**Figure 1:**
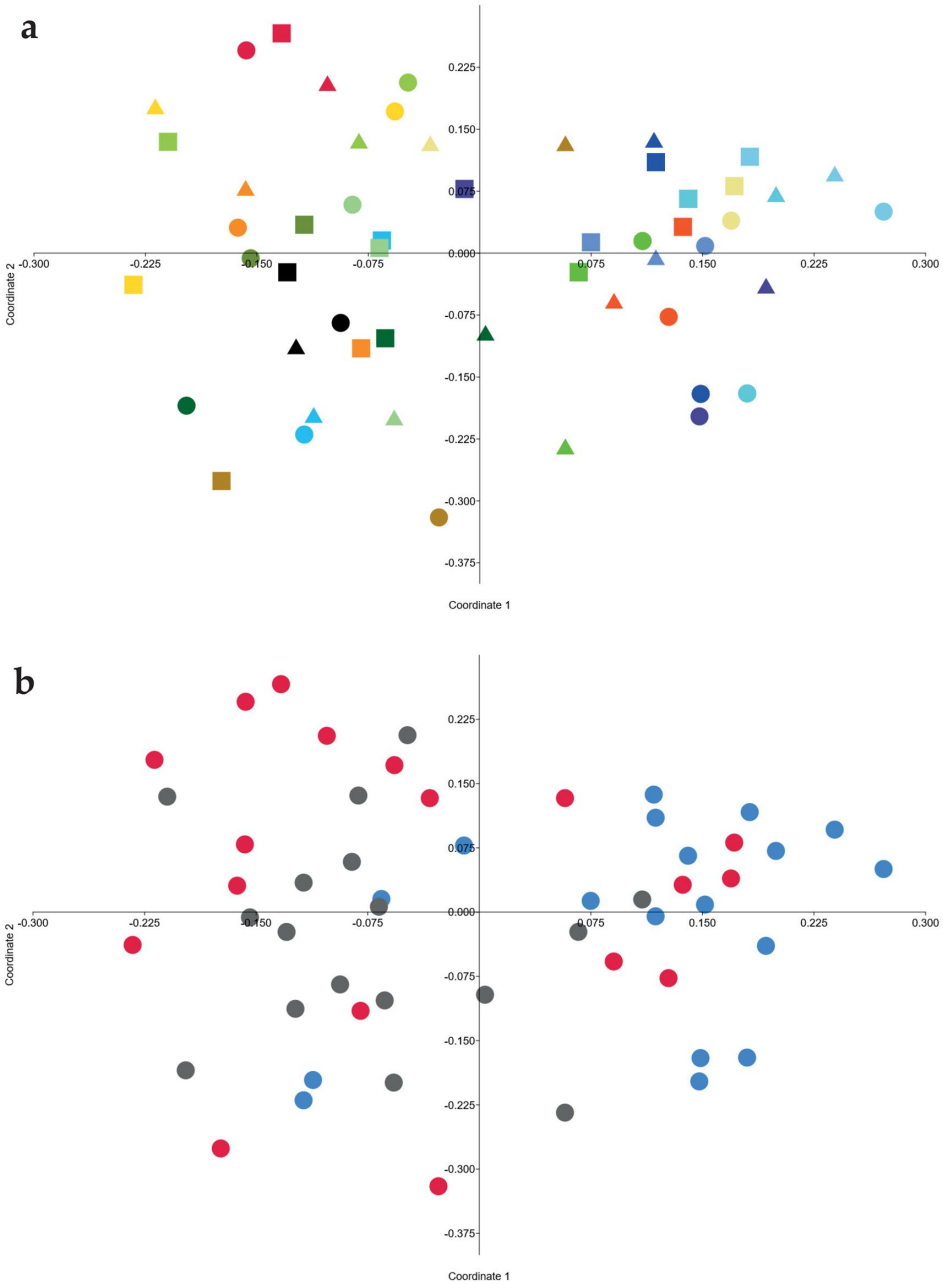
Principal coordinate analysis plots based on Bray Curtis distance metrics on operational taxonomic unit data. Each point represents one fecal sample from one dog following an experimental diet period. Both plots represent the same data, but colored differently. (**A**) Different colors represent individual dogs. Square = wheat diet (WHE), triangle = oat diet (OAT), dot = rye diet (RYE). (**B**) Different colors represent different diet orders. Blue = WHE–OAT-RYE, Red = OAT-RYE-WHE. Grey = RYE-WHE-OAT.

No clear effects of diet on general microbial composition in fecal samples were detected in PCoA analysis (Fig. 1a). Whether clustering could be explained by other traits was explored by coloring the samples in the PCoA plot by the dogs’ age, size, body condition score or gender. No such clustering was seen, but the samples from the group that started with WHE seemed to cluster separately from the groups starting with OAT or RYE (Fig. 1b).

There was an overall effect of diet on alpha diversity as measured by Shannon diversity index and PD whole tree (Table 3). Post hoc comparisons based on Shannon diversity and PD whole tree values revealed that microbial diversity was higher in samples collected after WHE compared with RYE (*p* = 0.011 and *p* = 0.012, respectively).

**Table 3:** Microbial diversity index (mean ± SD) for fecal samples collected from dogs following three experimental diets.

Diversity index	Wheat diet	Oat diet	Rye diet	*p*-value
Shannon	4.80 ± 0.45^a^	4.51 ± 0.43^ab^	4.45 ± 0.45^b^	0.011
Chao1	373 ± 93	412 ± 180	319 ± 69	0.058
PD whole tree	51.0 ± 16.6^a^	44.4 ± 12.8^ab^	38.8 ± 12.5^b^	0.004

^abc^Values within rows with different superscript letters are significantly different.

### Specific changes in fecal microbiota composition linked to the diet intervention

**Figure 2:**
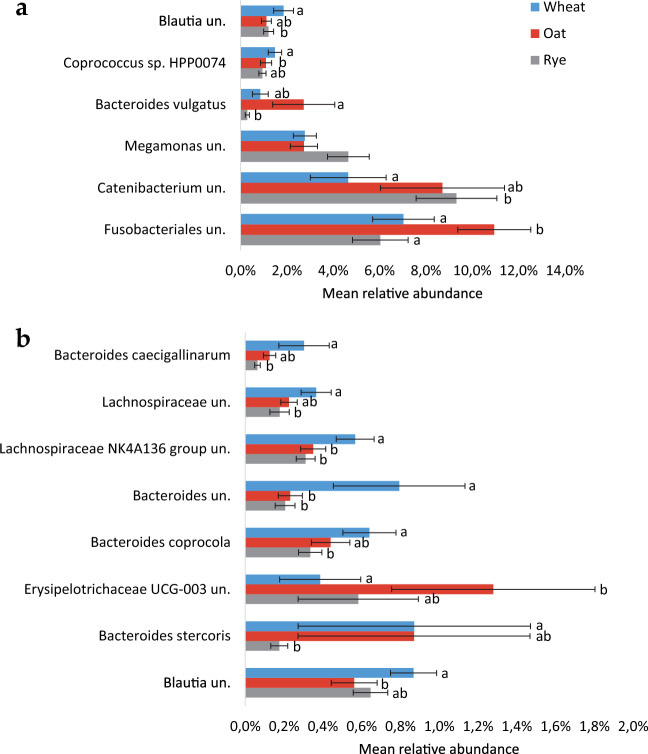
Relative abundance (mean ± SEM) of operational taxonomic units (OTU) with mean relative abundance > 0.1% and showing a significant difference in main treatment effect. Different letters within the same OTU indicate significant difference in log-transformed relative abundance between the different treatments. (**A**) OTUs with relative abundance > 1%; (**B**) OTUs with relative abundance between 1 and 0.1%.

**Figure 3:**
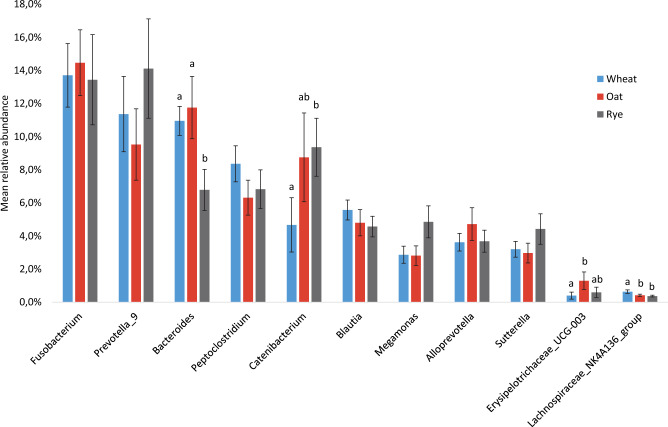
Relative abundance (mean ± SEM) of genera with mean relative abundance > 3% and/or differences due to a treatment effect. Different letters within the same genus indicate a significant difference between treatments.

Explorative univariate analysis of the dominating bacterial genera and OTUs indicated that diet had an effect on genus *Bacteroides*. In the analysis at OTU level, several OTUs classified to *Bacteroides* were least abundant in samples collected after RYE and most abundant in samples collected after WHE (Fig. 2). This was confirmed by the data at genus level, where *Bacteroides* was less abundant in samples collected after RYE than in samples collected after WHE (*p* = 0.004) or OAT (*p* = 0.014) (Fig. 3). There was also a trend for diet order to have an effect on genus *Bacteroides* (*p* = 0.066). Several OTUs belonging to family *Lachnospiraceae* were also least abundant in RYE samples and/or most abundant in WHE samples. Of the highly abundant genera, *Catenibacterium* was more abundant in RYE samples than in WHE samples (*p* = 0.026). Genus *Megamonas* was affected by diet (*p* = 0.046), but no effects could be confirmed in post hoc comparisons, although there was a trend for higher abundance of *Megamonas* in RYE samples compared with OAT samples (*p* = 0.057). Among the less abundant genera, *Lachnospiraceae*_NK4A136_group and *Erysipelotrichaceae*_UCG-003 were identified as being affected by diet (*p* = 0.008 and *p* = 0.014, respectively). There was a trend (*p* = 0.098) for a difference between diets in genus *Prevotella*_9, which was numerically most abundant in samples collected after RYE.

Diet order had a significant effect on genus *Prevotella*_9 (*p* = 0.0033), with the group of dogs that started with WHE having higher mean relative abundance of *Prevotella*_9 than the other two groups. Figures 2 and 3 show mean relative abundance of the significant OTUs and dominating genera, respectively. After FDR correction, one OTU belonging to family *Lachnospiraceae* was still significant, while the others were not (Supplementary Table S2).

### Short-chain fatty acids

**Figure 4:**
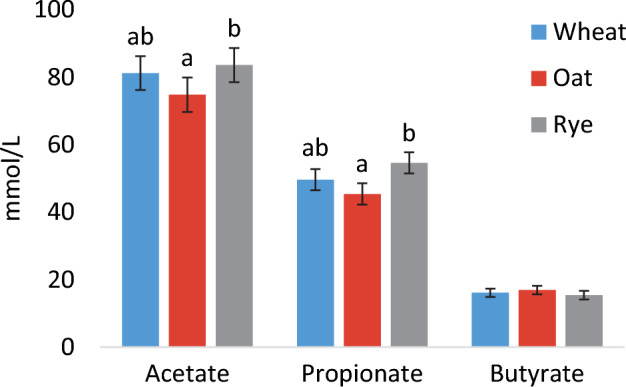
Fecal concentration (estimated marginal mean ± SEM) of acetate, propionate, and butyrate in dogs following consumption of the three experimental diets. Different letters indicate significant differences in concentration between treatments.

There was a trend for effect of diet on the concentration of total SCFA in fecal samples (*p* = 0.051) with estimated marginal mean ± SE for each diet: WHE: 147 ± 8, OAT: 138 ± 8 and RYE: 153 ± 8 mmol/L. When comparing the effect of diet on the three different SCFA there were significant differences in acetate and propionate with higher concentrations after RYE than OAT (*p* = 0.044 and *p* = 0.018, respectively) (Fig. 4). The relative proportions of the three SCFA in fecal samples did not differ between the diets.

## Discussion

This study explored the effects of dietary inclusion of three different types of whole grains (rye, oats or wheat) on fecal microbial composition and SCFA concentration in a population of privately owned dogs, acting as their own controls.

The PCoA analysis showed no clear effects of diet on general microbial community composition. For some dogs, samples from two of the different diet periods clustered together, but this pattern was not consistent for any specific diet. For a few dogs, all three samples were similar in composition, indicating an individual or environmental effect that could not be overruled by the diet. However, microbial alpha diversity (based on Shannon and PD whole tree values) was higher in samples collected after WHE than in samples collected after RYE. For all diets, Shannon diversity values were within the range previously reported for healthy dogs^19,45^. Previous studies comparing inclusion of whole grains in dog food are scarce. However, in studies on dogs, alpha and beta diversity have been reported to be unaffected both when comparing different ancient grains, one of which was oat groats^8^, and when comparing rye flour to fermented rye or cornmeal added to a vegetarian diet supplemented with feather meal^7^. Effects on fecal microbiota of consumption of whole-grain rye and wheat have been compared in a human study^26^, which found no significant effects on microbial alpha or beta diversity. In another human study comparing the same grains, some differences in general fecal microbial composition were observed^25^. Hence the effects of the grains are not clear. However, duration of the intervention and TDF intake varied between the different studies and these factors, together with large individual variation, make it difficult to draw any general conclusions about the effects on the overall microbial community in dogs and humans.

On investigating the effects on individual microbial genera and OTUs, we observed that genus *Bacteroides* and several OTUs belonging to that genus were least abundant in samples collected from dogs after RYE and highest after WHE. In a previous study by our research group, a decrease in *Bacteroides* after a diet with 50% rye inclusion, compared with samples collected before the diet period, was a factor of major importance for the difference in general microbial composition^21^. Decreased abundance of *Bacteroides* has been reported in studies on humans and pigs investigating the effects of rye kernel bread compared with refined wheat^22^ and an arabinoxylan-supplemented diet compared with a control diet^46^. A decrease in *Bacteroides* abundance in those studies was coupled with increased abundance of genus *Prevotella*. In the present study, *Prevotella* abundance was numerically highest after RYE and lowest after WHE but there was only a trend in the main diet effect (*p* = 0.098). Statistical significance was likely not reached due to large inter-individual variation, and larger sample size would perhaps confirm a significant difference between diets. In agreement with findings in the present study, our previous study showed significantly higher *Prevotella* abundance following the diet with highest inclusion of rye than following the wheat diet^21^. However, in that study whole-grain rye was compared to refined wheat and the dietary inclusion level of rye was higher (50% of DM).

The statistical model in the present study showed that diet order had an effect on relative abundance of genus *Prevotella*_9 (*p* = 0.033) and tended to have an impact on abundance of *Bacteroides* (*p* = 0.066). On reviewing the data, it emerged that there was a difference between groups in relative abundance already at the start of the study. This was also observed in the PCoA analysis, where the group that started with WHE clustered separately from the other groups. The group that started with WHE generally had lower *Bacteroides* abundance and higher abundance of *Prevotella*_9. In human studies, the concept of enterotypes has been used to divide people into groups with a gut microbiota dominated by either *Prevotella* or *Bacteroides*^47,48^. High *Prevotella*/*Bacteroides* ratio has been observed to have positive effects on glucose metabolism in human studies^49,50^. Whether the same is true for dogs has not yet been determined. Human studies have reported that these enterotypes have different fiber-fermenting characteristics and that the same fiber can benefit different bacteria depending on the enterotype^20^. It is not unlikely that these bacteria could have the same characteristics in dogs and that the individual responses observed in the present study were a result of this. There was over-representation of individuals with high *Prevotella*/*Bacteroides* ratio in the group that started with WHE. This could not have been predicted based on the data available prior to the study. In the ideal situation, we would have analyzed the fecal microbiota before grouping the dogs and taken microbial composition into consideration as a blocking factor. However, in the design used in the present study, the dogs were their own controls, so the initial clustering of dogs should be of minor importance.

*Catenibacterium* is a common bacterial member of the gut microbiota in dogs, yet often of relatively low abundance^45,51,52^. In the present study it was one of the most abundant genera, with highest abundance in samples collected after RYE. In contrast, in our previous study this taxon decreased in abundance from a baseline level on adding rye to the diet^21^. The reason for the discrepancy is unknown.

Of the less abundant OTUs, several belonging to family *Lachnospiraceae* showed significantly lower abundance after RYE compared with WHE. Similar results have been reported in a human study comparing microbial relative abundances after a rye diet with baseline values^25^. In that study, no such difference was observed after a whole-grain wheat diet.

It should be noted that this was an explorative study on the effects on fecal microbiota in a broad perspective, rather than an analysis of effects on specific bacteria. We therefore report both the uncorrected* p*-values and the *q*-values where a FDR correction was made to account for multiple statistical tests (Table S2). After FDR correction, the only remaining significant difference was for one OTU belonging to family *Lachnospiraceae*. This means that the uncorrected results should be interpreted with caution and that effects on specific taxa should be confirmed in further studies.

The lack of similar previous studies and the explorative nature of the study made it unfeasible to perform power calculations to determine sample size when planning the study. However, one study comparing other grains used 10 beagle dogs^8^ and other studies examining the effect of fiber on microbiota used 8–10 dogs^5,53^. Since we used privately owned dogs of different breeds living in a less controlled environment and with expected larger variation, we aimed to have around twice as many dogs as in those studies.

One possible limitation in the microbiota sampling was the time from defecation to placement in freezer and storage temperature. However, although there does not seem to be a consensus regarding best practice, several previous studies on the stability of microbiota samples have found no major differences when comparing different storage temperatures and duration from sampling to freezing^54–58^. In our study we sought to keep the time in room temperature to a minimum and the owners handled all samples from their dog equally, thus the effect of temperature should be the same for all samples from the corresponding dog and equal for all diets.

The concentrations of acetate and propionate were higher in samples collected after RYE compared with OAT. Similarly, in a previous study on pig fecal inoculum, production of acetate, but not production of propionate, was found to be higher from arabinoxylan substrate than from β-glucan^59^. In the present study, there was a trend for total SCFA concentration to be higher after RYE compared with OAT. Diet OAT had the highest inclusion of soluble dietary fiber, which was expected to make that diet more easily fermentable. However, it is plausible that digestion of oat fiber occurred more proximally in the intestine and thus the SCFA could also have been absorbed more proximally. A previous in vitro study with human inoculum showed faster fermentation rate of rye than oat samples^60^, although in that study only the bran of the grains was used, which means that the fiber content was likely higher than in our diets. Butyrate concentration did not differ between the three diets in the present study. In contrast, in a study in humans assessing the effects on fecal microbiota composition and function of whole-grain rye and wheat, higher production of butyrate after rye consumption was observed^25^. On comparing enterotypes, the same study found that propionate tended to be higher after rye consumption in a test group with *Prevotella* enterotype than in a test group with *Bacteroides* enterotype. In the present study, although there were differences in absolute levels of SCFA, the relative proportions of the three main SCFA were in line with previous reports in dogs^8^ and did not differ between the diets.

Using privately owned dogs provides an opportunity to investigate diet effects that are strong enough to have an impact on the nutrition of a broader dog population, but also poses challenges in terms of the less controlled environment than when using laboratory dogs. However, the dog owners participating in this study were likely more knowledgeable about the importance of following the instructions in an experimental research study than the average population, since they were staff and students at a university of agricultural sciences. Hence, the compliance was expected to be high, which was also indicated by the follow-up form in the end of each diet period. Moreover, the cross-over experimental design, in which the dogs serve as their own control, was another way of ensuring that potential differences in the studied effects would indeed be due to the differences in the diets and not in the environment or management. A few of the dogs received short term treatment with NSAID or topical ointment during the study When interpreting the results, those dogs did not show a deviating pattern during the medical treatment period compared to the other diet periods within the same dog.

## Conclusions

Inclusion of whole-grain rye, oats or wheat in the diet did not have clear differentiating effects on total fecal microbiota composition in dogs in this study. However, family *Lachnospiraceae* and genus *Bacteroides* were the gut microbial groups most affected by diet, with lowest relative abundance following consumption of the rye diet and a trend for a corresponding increase in genus *Prevotella*_9. This coincided with higher concentrations of acetate and propionate in fecal samples after consumption of the rye diet. Although rye had the largest impact on gut microbiota and SCFA, the relevance of the observed changes for the dog gut health need to be elucidated in future studies.

## Supplementary Information

Supplementary Tables.

## Data Availability

Sequence data have been deposited in the Sequence Read Archive (SRA) under accession number PRJNA933568. Data from digestibility and SCFA analyses and feed analysis can be obtained upon request from the corresponding author.
