# Supplementary material for: Effects of whole-grain cereals on fecal microbiota and short-chain fatty acids in dogs: a comparison of rye, oats and wheat
Source: Sci Rep. 2023 Jul 5;13:10920. doi: 10.1038/s41598-023-37975-4 (PMC10322915; doi:10.1038/s41598-023-37975-4)
Supplement: Supplementary file 1 — Supplementary Tables. [file 41598_2023_37975_MOESM1_ESM.pdf]

Effects of whole-grain cereals on fecal microbiota and short-chain fatty acids in dogs - A comparison of rye, oats and wheat

Hanna Palmqvist[1], Katja Höglund[2], Sara Ringmark[2], Torbjörn Lundh[1] and Johan Dicksved[1]

[1] Department of Animal Nutrition and Management, Faculty of Veterinary Medicine and Animal Science, Swedish University of Agricultural Sciences, Uppsala, Sweden

[2] Department of Anatomy, Physiology and Biochemistry, Faculty of Veterinary Medicine and Animal Science, Swedish University of Agricultural Sciences, Uppsala, Sweden

Supplementary Table S1: Demographics of dogs participating in an experimental study on diets containing whole grain of rye, oats or wheat.

Dog nr	Breed	Age (years)	Sex	Weight (kg)	BCS
1	Lagotto romagnolo	9.7	F	11.4	4
2	Lagotto romagnolo	5	F	11.2	5
3	Mixed breed	8.4	MC	41.5	5
4	Mixed breed	5.2	MC	8.7	6
5	Border Collie	5.2	F	15.2	5
6	Lhasa Apso	9.7	MC	8.5	6
7	Nederlandse Kooikerhondje	1.5	M	9.9	5
8	Poodle medium size	8.1	F	7.5	5
9	Australian Kelpie	4.5	MC	18.9	5
10	Weimaraner, short-haired	7.5	FS	25.1	6
11	Nova Scotia Duck Tolling Retriever	2.6	M	20.2	5
12	Mixed breed	3.2	MC	29	4
13	Whippet	3.6	FS	14.4	5
14	Samoyed	6.2	F	29.4	5
15	Mixed breed	7.2	F	30	6
16	Labrador Retriever	3	FS	19.4	5
17	Xoloitzcuintle	3.4	F	10	5
18	Mixed breed	8.4	FS	20.7	6

Sex: F= female, FS= female spayed, M= male, MC= male castrated. BCS= body condition score.

Supplementary Table S2. Relative abundance, in % (means ± SEM), of operational taxonomic units (OTU) with average relative abundance ≥ 0.1% and significant difference in main diet effect and genera with relative abundance ≥3% or significant difference in main diet effect.

				**Relative abundance**				
**Phylum**	**Family**	**Genus**	**Species**	**Wheat diet**	**Oat diet**	**Rye diet**	***p* diet effect**	***q*-value**
Firmicutes								
	Lachnospiraceae		Lachnospiraceae uncl.	0.37±0.1[a]	0.23±0.04[ab]	0.18±0.05[b]	0.0009	0.062
		Lachnospiraceae NK4A136 group		0.63±0.1[a]	0.41±0.1[b]	0.37±0.1[b]	0.008	0.16
			Lachnospiraceae NK4A136 group	0.57±0.1[a]	0.35±0.1[b]	0.31±0.05[b]	0.005	0.14
			Coprococcus sp. HPP0074	1.5±0.3[a]	1.1±0.2[ab]	0.95±0.2[b]	0.028	0.20
		Blautia		5.6±0.6	4.8±0.8	4.6±0.6	0.10	0.22
			Blautia uncl.	1.9±0.4[a]	1.1±0.2[b]	1.2±0.2[ab]	0.013	0.15
			Blautia uncl.	0.87±0.1[a]	0.56±0.1[b]	0.65±0.1[ab]	0.035	0.20
	Veillonellaceae	Megamonas		2.9±0.5	2.8±0.6	4.9±1.0	0.046	0.22
			Megamonas uncl.	2.8±0.5	2.7±0.6	4.7±0.9	0.044	0.22
	Erysipelotrichaceae	Erysipelotrichaceae UCG-003		0.39±0.2[a]	1.3±0.5[b]	0.59±0.3[ab]	0.014	0.19
			Erysipelotrichaceae UCG-003 uncl.	0.39±0.2[a]	1.3±0.5[b]	0.58±0.3[ab]	0.015	0.15
		Catenibacterium		4.7±1.6[a]	8.7±2.7[ab]	9.4±1.8[b]	0.032	0.22
			Catenibacterium uncl.	4.6±1.6[a]	8.7±2.7[ab]	9.3±1.7[b]	0.033	0.20
	Peptostreptococcaceae	Peptoclostridium		8.4±1.1	6.3±1.1	6.8±1.2	0.08	0.22
Bacteroidetes								
	Bacteroidaceae	Bacteroides		11.0±0.9[a]	11.8±1.9[a]	6.8±1.2[b]	0.003	0.11
			Bacteroides uncl.	0.79±0.3[a]	0.23±0.1[b]	0.21±0.1[b]	0.006	0.14
			Bacteroides coprocola	0.64±0.1[a]	0.44±0.1[ab]	0.33±0.1[b]	0.014	0.15
			Bacteroides stercoris	0.87±0.6[a]	0.87±0.6[ab]	0.18±0.04[b]	0.026	0.20
			Bacteroides vulgatus	0.85±0.3[ab]	2.7±1.3[a]	0.29±0.1[b]	0.031	0.20
			Bacteroides caecigallinarum	0.30±0.1[a]	0.13±0.03[ab]	0.06±0.01[b]	0.04	0.21
	Prevotellaceae	Prevotella_9		11.4±2.3	9.5±2.2	14.1±3.0	0.098	0.22

		Alloprevotella		3.6±0.5	4.7±1.0	3.7±0.7	0.81	0.93
Fusobacteria								
	Fusobacteriaceae	Fusobacterium		13.7±1.9	14.5±2.0	13.4±2.7	0.58	0.70
			Fusobacteriales uncl.	7.0±1.3^{a}	10.9±1.6^{b}	6.0±1.2^{a}	0.008	0.14
Proteobacteria								
	Alcaligenaceae	Sutterella		3.2±0.5	3.0±0.6	4.4±0.9	0.20	0.34

Values on the same row without common superscript letters differ significantly ($p < 0.05$) in post-hoc pairwise comparisons. *q*-value obtained from correction with false discovery rate (FDR) according to Benjamini-Hochberg.
